# Retinal Hyperreflective Foci Are Biomarkers of Ocular Disease: A Scoping Review With Evidence From Humans and Insights From Animal Models

**DOI:** 10.1155/joph/9573587

**Published:** 2025-05-29

**Authors:** Mohd N. Mat Nor, Colin R. Green, David Squirrell, Monica L. Acosta

**Affiliations:** ^1^School of Optometry and Vision Science, The University of Auckland, Auckland, New Zealand; ^2^Department of Anatomy and Physiology, Faculty of Medicine, Universiti Sultan Zainal Abidin, Kuala Terengganu, Malaysia; ^3^Department of Ophthalmology, The University of Auckland, Auckland, New Zealand; ^4^Auckland District Health Board, Auckland, New Zealand; ^5^New Zealand National Eye Centre, The University of Auckland, Auckland, New Zealand; ^6^Centre for Brain Research, The University of Auckland, Auckland, New Zealand

**Keywords:** age-related macular degeneration, diabetic retinopathy, glaucoma, optical coherence tomography, retinal hyperreflective foci

## Abstract

**Background:** Abnormalities in the retina have a profound impact on vision, and accurate diagnosis and monitoring are essential for effective clinical management. Retinal hyperreflective foci (HRF), lesions, or dots, identified using optical coherence tomography (OCT), are observed in both animals and humans and have been associated with several ocular conditions, including diabetic retinopathy (DR), age-related macular degeneration (AMD), and retinal vascular diseases.

**Methods:** To evaluate the relevance of retinal HRF, we conducted a comprehensive scoping review of the literature published up to July 2024 including in the discussion key papers that emerged in 2025. Our search spanned electronic databases utilizing carefully identified search terms related to HRF and OCT within the last six years. We excluded publications on HRF outside the retina, treatments, non-peer-reviewed content, duplicates, studies older than 6 years, and those not focused on AMD, DR, or glaucoma.

**Results:** A total of 141,085 records were initially identified from various databases and further refined based on keywords and content relevance. Finally, 42 reports meeting the criteria were retained for in-depth analysis. HRF were observed mainly in OCT scans of the AMD retina, as well as in DR and, to a lesser extent, in other retinopathies and interestingly in glaucoma. In AMD, HRF are described as a marker for disease progression, often associated with a compromised photoreceptor structure. In DR, HRF indicated issues such as abnormal blood vessels and cellular changes linked to microglia activation. In glaucoma, HRF may reflect microglia and macrophage activation. Most publications concur that the presence of HRF correlates with inflammatory processes and aging in the retina, with early appearance of small HRF serving as a biomarker for ocular disease. The size of HRF and their location were consistent with disease presentation.

**Conclusion:** There is an agreement that HRF of less than 30 μm are biomarkers of inflammation in the retina despite having variable intraretinal locations. HRF resulting from the effect of aging can be discerned from AMD based on their quantity and appearance. The results show the importance of HRF as a biomarker of ocular disease and confirm that HRF are indicative of an inflammatory eye disorder.

## 1. Introduction

The retina is a complex and vital component of the human visual system, playing a central role in vision. However, over time, the retina is prone to damage from various factors such as prolonged exposure to systemic conditions, genetic disorders, and age-related degeneration. Research has demonstrated that structural damage in the retinal layers can be detected before visual symptoms of the pathology manifest. Retinal hyperreflective foci (HRF) on optical coherence tomography (OCT) images have emerged as a likely novel biomarker of retinal inflammation and vascular damage.

Although the abbreviation HRF is most commonly used to refer to “hyperreflective foci,” some publications also refer to them as “hyperreflective retinal foci.” Additionally, in a broader medical literature, HRF may describe hyperreflective dots or lesions in scans of other organs. However, in the context of ophthalmology, we use HRF exclusively to denote HRF—small, discrete reflective spots observed in OCT scans of the retina. For clarity and consistency, we will use the term HRF throughout this paper.

Most publications found the presence of HRF in age-related macular degeneration (AMD) and diabetic retinopathy (DR), but they have also been observed in other conditions such as retinal vein occlusion, retinal edema, macular telangiectasia, and retinitis pigmentosa [1–6]. Additionally, HRF have been reported, though less frequently, in other ocular diseases such as Fabry's disease and Stargardt's disease [4, 7, 8]. We also focused on the presence of HRF in glaucoma, as these have been less extensively studied. According to the World Health Organization (WHO) [9], AMD, DR, and glaucoma are among the top five causes of vision impairment worldwide, with reliable biomarkers for detecting the earliest stages of these diseases still lacking. This scoping review aims to map the existing literature on HRF associated with these three ocular conditions, assess their potential as biomarkers, and clarify key concepts that link HRF to disease.

HRFs are easily detected during routine retinal imaging using OCT, hence allowing early identification of underlying structural and cellular changes. As a noninvasive biomarker, HRF can be continuously monitored to evaluate ocular disease-related complications [10–14]. The structural characteristics and specific locations of HRF are crucial for interpreting their significance in various pathological conditions [1, 2, 15]. HRF are particularly valuable for identifying early signs of disease, guiding interventions to slow disease progression, and monitoring responses to treatment [16, 17].

Given the growing recognition of HRF as a biomarker for ocular diseases, we have conducted a scoping review of the literature describing the expression and appearance of HRF in various ocular conditions and here discuss the findings.

## 2. Methods

To assess the significance of HRF in OCT images, we conducted a scoping review of the relevant literature available up to July 2024. We searched electronic databases, using predefined keywords to establish inclusion and exclusion criteria. We included studies that investigated the pathophysiology and clinical features of ocular diseases associated with HRF.

### 2.1. Inclusion Criteria

We focused on publications that provided relevant data sets and those discussing HRF and their association with eye diseases such as the distribution and prevalence of HRF in AMD, DR, and glaucoma. These diseases were selected based on the WHO's list of the top causes of vision impairment affecting the retina [10]. The key search terms used in the inclusion criteria were related to “age-related macular degeneration,” “diabetic retinopathy,” “hyperreflective foci,” “hyperreflective dots,” “hyperreflective spots,” “microaneurysm,” “optical coherence tomography,” “retina,” and “human” or “animal” within the last 6 years. We included publications including the keyword ‘HRF' and ‘glaucoma', although these were less significantly associated with pathology.

### 2.2. Exclusion Criteria

We excluded publications that focused on HRF outside the eye, as well as articles that primarily discussed intravitreal treatment with antivascular endothelial growth factor (VEGF) injections for AMD and DR. This is because anti-VEGF treatment reduces proinflammatory cytokines and endothelial cell permeability, and consequently, HRF often regresses after treatment. Publications were also excluded for the following reasons: they were not peer-reviewed articles or scientific material, the title did not align with the inclusion criteria, the publication was beyond 6 years, they were duplicates, there was only an abstract available, or they were not relevant to the pathologies we targeted. Duplicate records across databases, review articles, and abstracts were also removed. Additionally, studies involving diseases other than AMD, DR, or glaucoma, those reporting HRF only in the vitreous, or articles that had insufficient content to meet our criteria were excluded.

### 2.3. Information Sources

Sources included peer-reviewed academic journals, recognized sector databases, published conference presentations, dissertations/theses, empirical studies, government reports, and reports from other organizations, along with historical records from PubMed, Science Direct, and Google Scholar from 2019 to 2024.

## 3. Results

**Figure 1:**
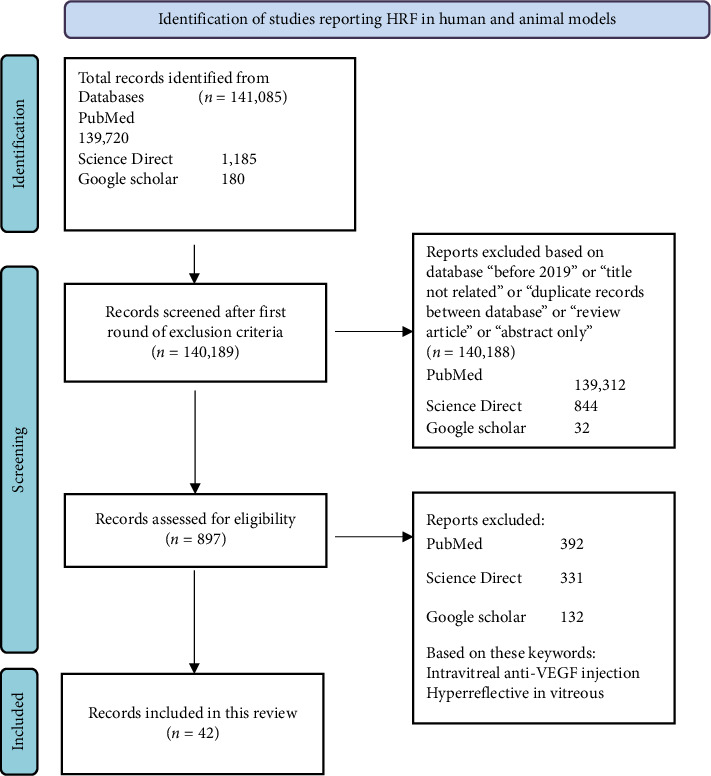
Inclusion and exclusion criteria of the literature using the PRISMA flowchart [18].

To report on the importance of HRF as biomarkers of retinal disease, the number of publications referring to HRF in the retina in humans and other animals was counted, and scoping review guidelines were used (Figure 1). A total of 141,085 records were identified from the various databases searched. After applying inclusion and exclusion criteria, 42 reports were retained, and they are discussed below and in the discussion.

**Figure 2:**
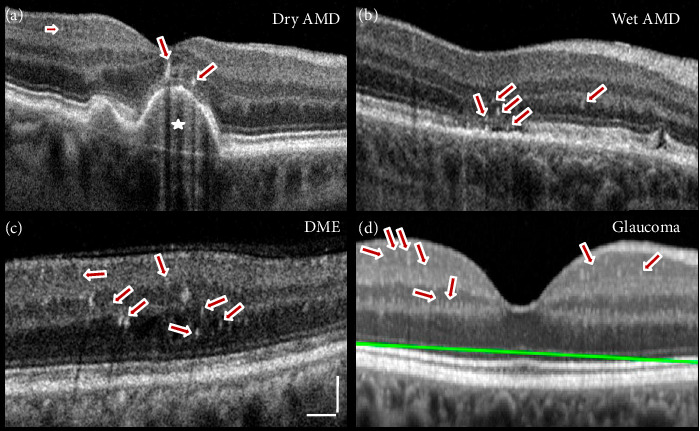
. HRF appearance in AMD, DME, and glaucoma. (a) HRF (arrows) often appear near the RPE layer, especially in association with drusen (star) or subretinal deposits in dry AMD. (b) HRF are more clustered, particularly in the outer retinal layers, in advanced AMD. (c) Clusters of HRF in this region are commonly linked to macular edema. In the INL, the presence of HRF is consistent with retinal edema and vascular leakage, while scattered HRF in the ONL is indicative of fluid accumulation. Scale bar equals 100 μm. (d) HRF are present in the GCL, INL, and IPL, potentially indicating parainflammatory processes or structural damage in glaucoma. The green line represents the RPE axis. (a)–(c) obtained by Dr. David Squirrell. (d) A comparison of hyper-reflective retinal spot counts in optical coherence tomography images from glaucomatous and healthy eyes. J Clin Med. 2021 10(20): 4668.

The literature shows that the term HRF refers to a biological event characterized by dot-like or circular lesions observed within retina, vitreous, and choroid using noninvasive OCT imaging [1, 19]. The significance of HRF in various pathological conditions is influenced by their structural features and topographic location. Specifically, HRF less than 30 μm in diameter and with certain reflectivity levels provide valuable insights as biomarkers [1, 15]. In contrast, microaneurysms are larger and confined to the vascular layers of the retina. Histological analysis has shown that HRF may result from migrating retinal pigment epithelium (RPE) cells, increased macrophage activity, microglial activation, proteinaceous leaks, or lipid accumulation [16, 20, 21]. In some cases, HRF have been confirmed as tiny clusters of activated microglia cells migrating from the inner retinal layers (INLs) to the outer layer. HRF in AMD are typically found in the outer retina, particularly in the subretinal space and outer nuclear layer (ONL), where they are associated with changes in the RPE and the presence of drusen; a higher density of HRF correlates with advanced disease stages and potential visual acuity decline. In contrast, HRF in DR are predominantly located in the inner retina, particularly in the INL, and are associated with retinal vascular structures, reflecting conditions such as intraretinal edema, ischemia, or microaneurysms [1, 2, 15, 22–25]. The literature confirms that there is a pattern in the presentation of HRF in each condition, and the summary of HRF size and location for each disease is outlined in Table 1.  Figure 2 illustrates the location of HRF in the human pathologies, providing a visual representation of their distribution in AMD, DR, and glaucoma.

The prognosis of retinal diseases is influenced by multiple factors, including the specific retinal layer involved and the degree of hyperreflectivity observed. In AMD, the presence of HRF in the subretinal space or inner retina is associated with more severe disease progression and a higher risk of vision loss, especially in cases of geographic atrophy (GA) or neovascular AMD. In macular edema, regardless of its etiology, HRF in the INLs, especially within cystic spaces, can indicate chronicity and a poor response to treatment. Studies indicate that a higher density of HRF (e.g., more than 20 HRF per 1 mm^2^) can predict progression to GA within 2–5 years in AMD [26, 27]. Additionally, clusters of more than 10 HRF per lesion are linked to subretinal fibrosis and vision loss within 1–3 years, further supporting HRF's role as a prognostic biomarker in AMD [27]. Moreover, HRF in the central area, particularly when exceeding 15 foci, has been found to correlate with persistent edema and worsening visual acuity over 1-2 years, highlighting its value in monitoring macular edema [4, 20]. Similarly, in DR, the detection of HRF in the INLs correlates with microglial activation or inflammatory cell presence, which suggests a greater likelihood of developing diabetic macular edema (DME) or proliferative diabetic retinopathy (PDR). In glaucomatous eyes, an HRF density greater than 10 foci per scan is associated with progressive retinal layer thinning and visual field loss within 2–4 years [5, 29], demonstrating the utility of HRF in tracking disease progression in glaucoma.

**Figure 3:**
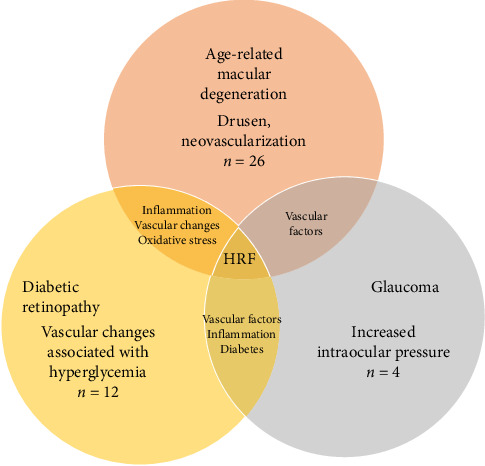
Venn diagram illustrating the overlap of key characteristics observed in age-related macular degeneration, diabetic retinopathy, and glaucoma through hyperreflective foci in OCT. The largest section of the literature (*n* = 26) indicates the presence of drusen and changes in retinal vasculature as key features visible in early or late stages in patients with AMD. Vascular changes are the defining feature in diabetic retinopathy (*n* = 12). Glaucoma, depicted with a smaller number (*n* = 4), is characterized by increased intraocular pressure, which leads to optic nerve damage. Changes related to glaucoma may also show up as HRF in OCT scans. The diagram shows underlying mechanisms where these conditions overlap, suggesting that some patients may exhibit signs of multiple conditions, particularly in the context of HRF in OCT imaging. HRF are a common component across these three major diseases.

We further grouped the scientific literature to quantify references to HRF in the context of each specific ocular pathology. Our findings revealed that HRF were mentioned in 26 publications pertaining to AMD, 12 publications related to DR, and 4 publications concerning glaucoma. Remarkably, our analysis found that 20 publications across all examined pathologies explored the correlation between HRF and inflammatory processes within the retina (Figure 3).

## 4. Discussion

Our exploration of the literature related to HRF association with pathophysiological mechanisms underlying key ocular disease type has shown that retinal hyperreflective spots, dots, and foci are consistently biomarkers of the disease. The formation of HRF is a complex process, and their hyperreflectivity may vary depending on the specific retinal pathology or disease. However, multiple studies have identified common mechanisms across diseases that contribute to the formation of HRF.

Beyond the eye, HRF can manifest in various organs. For instance, in the brain, these foci may indicate lesions or areas of heightened signal intensity, often associated with conditions such as multiple sclerosis or small vessel ischemic disease [30, 31]. HRF have also appeared in echocardiograms and cardiac MRI scans, potentially signaling fibrosis, scarring, or deposits in the heart muscle [32]. Additionally, HRF have been linked to cysts, tumors, lesions, or other anomalies that affect tissue density or integrity in the kidneys, liver, and lungs [33–35]. Unlike in the retina, the significance of HRF in other organs can vary widely based on the context and specific characteristics observed in imaging studies. The characterization and correlation of HRF across these three retinal diseases are of increasing interest, with publications indicating a common HRF component [5, 25, 36].

### 4.1. HRF in AMD

Within the AMD category, 14 publications specifically discussed HRF in the inner retina. In one study, HRF were categorized as either manifestations of pigment granules outside cells and debris from outer segments (referred to as outer HRF) or as shifting and clustering of deteriorated RPE cells or microglia (referred to as inner HRF) [26]. Additionally, HRF were categorized based on observations of at least one distinct or two or more ambiguous focal, discrete, well-defined punctate lesions exhibiting reflectivity equal to or exceeding that of the RPE within the neurosensory retina [35]. The use of HRF as a biomarker for AMD is also supported by data indicating a particularly strong correlation between the disease state and intraretinal HRF [37].

AMD research often subdivides the disease into early, intermediate, and late stages, with varying explanations for HRF at each stage. In dry AMD, these lesions are commonly located above or adjacent to drusen and are not associated with intraretinal blood vessels, unless damaged, according to prior definitions [1, 28]. More precisely, a statistically significant direct relationship between drusen and HRF has been observed [37, 38]. Inflammation is also linked with HRF, which is central in AMD development [1, 2, 22–24, 38, 39]. In dry AMD, it is postulated that HRF likely represent migrating RPE cells, which may move along the primary orientation of retinal axons, particularly the fibers of the Henle's layer. Since these fibers are oriented centripetally around the foveal center, their alignment could result in an accumulation of cells near the fovea [2]. In other studies of dry AMD, HRF were found more frequently in the superior and temporal areas of the retina [40]. However, in advanced stages of the disease such as in macular atrophy, HRF appeared without any connection to drusen. Whilst early-stage AMD is often asymptomatic and linked to exposure to environmental and systemic factors, it can progress to later stages, with the overall HRF presence showing a positive correlation with age [3]. In these AMD cases, HRF were unevenly distributed across the retina but were more frequently found on the temporal side of the fovea [2] with a predominant presence at the fovea [41]. HRF have also been associated with exudation [28], with high concentrations seen in the junctional zone of GA [42]. HRF as a biomarker of progress to GA are related to the earliest changes in OCT structural characteristics linked to microvascular lesions [43]. The volumes of intraretinal cystoid fluid, subretinal fluid, pigment epithelial detachment, and subretinal HRF material were found to be significantly increased at the onset of exudation, accompanied by structural changes such as HRF and loss of integrity in the ellipsoid zone (EZ) and the external limiting membrane (ELM). These HRF visible on OCT may signal cellular infiltration and are characteristics of disease progression in neovascular AMD [44].

Additionally, HRF have been associated with the compromised photoreceptor structure, where HRF preceded irregularities and reductions in outer segment length [45]. This was seen as disturbances in the EZ band and the emergence of large HRF that aligned with segmented ellipsoids. In that study, early signs of AMD correlated with the presence of HRF and a hazy appearance of the eye [45]. Alongside structural changes, HRF are also related to retinal dysfunction with progressive HRF associated with reduced amplitude in scotopic and photopic electroretinography responses [46]. Areas of the retina with HRF compromised retinal sensitivity and mean sensitivity thresholds [47], and HRF have been linked to delays in dark adaptation, which may indicate impaired visual cycle function [24]. Recent reports indicate that HRF in the choroid are more concentrated in the central macular region compared to pericentral or peripheral macular area [48]. Furthermore, studies suggest that patients with intermediate AMD showing persistent HRF may be at higher risk for disease progression, as HRF can indicate the possible emergence of choroidal hypertransmission defects [36]. While HRF in the choroid are not seen in early stages of AMD, in patients with GA, OCT images of the choroid show HRF of variable sizes and shapes near Bruch's membrane or the edges of the blood vessels but not inside the vessels [49]. Chhablani et al. has also observed these HRF in the choroid of advanced retinitis pigmentosa and Stargardt disease patients. Altogether, these observations indicate that HRF in the choroid are the consequence of pathology rather than biomarkers of the progression of disease [50].

### 4.2. HRF in DR

Among pioneering studies, the work of Vujosevic and Midena in 2013 has received considerable interest for supporting HRF as a biomarker in diabetic retina where inflammation plays a role [51]. Similarly to other vascular conditions, in DR, HRF are indicative of microvascular abnormalities and neurodegenerative changes in the retina, potentially highlighting regions of ischemic insult or inflammation due to hyperglycemia [6, 16]. In patients with DME, the presence of HRF observed using spectral-domain optical coherence tomography (SD-OCT) was more common compared to patients with branch retinal vein occlusion-macular edema, central retinal vein occlusion-macular edema, or uveitic macular edema [4]. DME is associated with the build-up of excess fluid in the extracellular space, specifically in the macular region, and the appearance of HRF was linked to the frequency of hard exudates [4] and the progression of DR [16] and may be due to hemorrhage in the inner nuclear and outer nuclear plexiform layers of the retina [48]. Moreover, in patients with DME, a decline in visual acuity negatively correlated with an increase in HRF and the volume of subretinal fluid [52]. These HRF seem to be early indicators appearing shortly after exudate accumulations in the retina if there is recruitment of macrophages. They have a role in the phagocytosis of leaked lipids or proteins, a process that precedes the development of clinically detectable hard exudates [1, 16].

In an animal model of DR, macroaneurysms, microaneurysms, and small HRF were observed in OCT images across all retinal layers, particularly in the inner nuclear layer and ONL. Further analysis indicated that these small HRF are early signs of microglia activation, and a reduction in retinal electrical activity, as measured by focal electroretinogram (fERG), was noted in areas where HRF were present [15]. It has been proposed that HRF seen in DR represent either hard exudates or microglia associated with inflammation [15, 20]. More recent studies conclude that the increased number of HRF in DME and the formation of subretinal fluid support a role for microglia in the dysfunction of the RPE [53]. In another animal model of DR, discrete retinal areas containing HRF were analyzed using fERG, confirming that HRF are associated with decreased ERG amplitude [52]. They are similarly associated with reduced visual acuity in patients [54]. Additionally, in a mouse model of DR treated with the connexin hemichannel blocker Peptide5, a reduction in retinal HRF was observed, correlating with inhibited microglial infiltration and decreased inflammasome activation [18]. A study involving 60 patients with DME reported that HRF within the choroid were observed concomitantly with a disrupted ELM and EZ, suggesting that the disrupted retina allowed the migration of these HRF into the choroid. The authors also hypothesized that an increasing number of HRF predicts greater severity of DR and worse visual acuity. Progression to PDR was associated with an increasing number of HRF in the choroid [55]. The relevance of HRF in DR is supported by emerging HRF segmentation algorithms that assist ophthalmologists in the early diagnosis of DR [56].

### 4.3. HRF in Glaucoma

Although HRF have also been observed in some cases of glaucoma, their role as biomarkers in this context remains less well-established compared to AMD and DR, owing to the limited number of studies specifically addressing this association. Existing evidence suggests that they may reflect localized retinal nerve fiber layer thinning or damage associated with elevated intraocular pressure, or they could represent inflammatory responses to pressure on the retina [5, 57]. The role of HRF as a biomarker in glaucoma is less well-supported, as fewer studies have investigated this association. However, in an OCT analysis of patients with glaucoma, the number of HRF was higher compared to controls, leading authors to propose that their presence may indicate neuroinflammation in the retina [5]. In OCT en-face images, hyperreflective structures were presumed to be activated retinal astrocytes and Müller cells, positively correlated with aging and localized in the RNFL. They were bigger than HRF, and the authors concluded due to variability in the presentation of these hyperreflective structures in OCT en‐face imaging that they did not consider them to be indicators of glaucoma progression [58]. Moreover, studies of the hyperreflectivity at the border of the vitreous and retina identified that the presence of small changes in opacity (< 10 μm^2^) was indicative of changes in homeostasis in glaucoma, signaling a connection between HRF and immune cells, particularly microglia and macrophages [29]. In another study of glaucomatous eyes, 31 out of 34 patients with advanced glaucomatous optic neuropathy presented with HRF along with thinning of the ganglion cell and inner plexiform layers [57]. These findings, while preliminary, suggest a possible link between HRF and glial or immune cell activation in glaucomatous damage. Given the scarcity and variability of current evidence, further studies, particularly those integrating imaging with histological and immunohistochemical analyses in both human and experimental models, are needed to clarify whether HRF in glaucoma reflect disease-specific pathology or broader neurodegenerative processes. Until then, interpretations of HRF in glaucoma should remain cautious and context-dependent as discussed in the literature [5, 29, 57, 58].

### 4.4. HRF is Associated With Molecular Inflammation

In the retina, most publications concur that the presence of HRF correlates with inflammatory processes [1, 2, 4–6, 15–17, 19, 22–25, 36, 38, 48, 59–62]. Certainly, inflammation is recognized as an early contributor to the development and progression of AMD, DR, and glaucoma. Inflammatory and cellular mediators can induce cellular damage, alter the integrity of the blood-retinal barrier, and promote the migration of immune cells into the retina [58]. In relation to inflammatory cells, HRF primarily correspond to the activation of microglial cells in the retina, and there appears to be a correlation between the presence of HRF and cytokines produced by monocytes and microglia in the central nervous system [15, 16, 25]. These studies have highlighted the role of inflammation-related molecules and various immune cells, such as macrophages, dendritic cells, neutrophils, T lymphocytes, and B lymphocytes, which are associated with both innate and adaptive immune responses and can contribute to hyperreflectivity as they move through the retina [15, 25, 36, 63–65].

### 4.5. HRF in the Aging Retina

Although most researchers suggest that the presence of HRF is indicative of disease, alternative interpretations have been proposed. HRF can be found in both normal inner and outer retina although it is of note that their persistence over time is associated with a less favorable visual outcome for patients [66]. Corradetti et al. proposed that HRF may represent normal anatomical landmarks in the retina, particularly Müller cell end feet or the basal lamina of the inner limiting membrane [67]. This group used en-face SD-OCT combined with confocal analysis to produce transverse images of retinal and choroidal layers at specified depths. Their study evaluated normal eyes in healthy adults up to 90 years of age. Consistent with other published data, they found a positive correlation between the emergence of HRF and aging. Specifically, HRF numbers increased in participants aged 50 years and above. In OCT-B images, the HRF displayed an elevated or flattened linear morphology that made them substantially different from the HRF reported in pathology. Although they were unable to conclusively demonstrate that HRF progression was related to disease progression, the authors suggested that their findings were likely due to early-stage Müller and microglial cell activation, as microglia can invade inner nuclear cells up to the photoreceptor layer, leading to photoreceptor damage [16, 26, 68]. In contrast, another study examining white lesions in the retina and choroid described HRF as benign, self-limiting phenomena, particularly in the context of autoimmune disease [59]. With advancing age, the retina undergoes a range of physiological changes that may contribute to the appearance of HRF distinct from those seen in disease. These include the gradual accumulation of metabolic by-products, subtle alterations in the Müller cell structure, and increased microglial surveillance activity [69]. Aging is also associated with mild gliosis, leading to changes in the reflectivity of the retinal layers and a decline in phagocytic efficiency, all of which can give rise to hyperreflective elements on OCT imaging. This highlights the importance of considering patient age, retinal location, and temporal persistence when interpreting HRF to better distinguish physiological changes from early signs of disease. The classification system we show in Table 1 accounting for morphology, location, and patient context is necessary to distinguish between pathogenic and nonpathogenic HRF.

Discerning HRF due to normal low-level inflammation associated with aging from those related to AMD relies on combining multimodal imaging, clinical history, and functional testing to more accurately distinguish HRF due to aging from those associated with AMD. HRF associated with normal aging tend to be sparse and scattered, flat, and in the inner limiting membrane, often confined to the INLs without significant clustering [24]. In contrast, HRF in AMD typically appear in the RPE or near drusen, concentrated around areas of retinal atrophy, subretinal fluid, or choroidal neovascularisation, indicating ongoing disease processes. As described by Wu et al., HRF associated with aging remain relatively stable over time, whereas HRF in AMD may increase in size or number or migrate toward the RPE [66]. HRF in AMD are often already linked to visual impairment due to their association with structural damage in the macula and may represent activated microglia, lipid-laden macrophages, or lipoprotein deposits, often indicating a higher inflammatory component. A history of AMD or a family history of the disease increases the likelihood that HRF are related to AMD. However, these do not account for the prolonged effects of inflammation associated with HRF in various disease states.

This clinical context highlights the importance of cautious interpretation in both diagnostic and prognostic settings. Clinically, the assumption that all HRF indicate pathology may lead to overdiagnosis or misclassification, especially in older patients or those with coexisting systemic conditions such as autoimmune diseases. Conversely, overlooking subtle HRF changes in early disease could delay intervention. Standardized imaging criteria and longitudinal studies that correlate HRF characteristics with histopathology and functional outcomes are needed. From a research perspective, future studies should aim to stratify HRF by origin, such as microglial versus lipoprotein-derived, and track their evolution across disease stages and treatment responses. HRF observed in animal models [15], where cellular sources and mechanisms can be directly assessed, may help clarify current ambiguities and support a more precise classification of HRF in human retinal disease.

## 5. Conclusions

This scoping review highlights the clear link between inflammation, HRF, and disease progression in AMD, DR, and glaucoma. HRF observed in OCT images serve as valuable biomarkers for diagnosing, monitoring, and predicting the progression of retinal diseases. The findings reveal a common feature across these conditions manifested as HRF, enhancing our understanding of their pathophysiology. However, further research, particularly in animal models and human tissue samples, is essential to address the variability of HRF that could better explain their underlying pathological mechanisms and relationship to disease progression.

## Figures and Tables

**Table 1:** Descriptors of HRF in AMD, DR, and glaucoma that can be used as biomarkers of disease progress.

Condition	Size	Location	Proposed origin	Appearance	Reflectivity	Prognosis	Reference
Dry AMD	< 30 μm	Outer retina and around drusen	Activated microglia or degenerated RPE cells	Dot-like or round lesions	Comparable to RPE	A higher density (> 20 HRF in 1 mm^2^) predicts progression to GA over 2–5 years	Flores et al. [26] Wakatsuki et al. [27]

Neovascular AMD	< 30 μm in clusters	Inner retina	Activated microglia	Well-defined small lesions	With shadows	Clusters of > 10 HRF per lesion are linked to subretinal fibrosis and vision loss within 1–3 years	Nawash et al. [28]

Diabetic macular edema	< 30 μm	INL, ONL, IPL, and OPL	Microglial cells responding to ischemia and inflammation	Discrete, typically dot-like or round, clear bounders	≥ RPE not visible in color images or infrared	> 15 HRF in the central area correlate with persistent edema and worsening visual acuity over 1-2 years	Zhu et al. [4] Yao et al. [20]

Glaucoma	< 30 μm	Central retina and all layers except ONL and RPE	Activated retinal microglial cells	Isolated, punctiform, no back shadowing	Similar to RNFL	Increased HRF density (> 10 HRF per scan) is associated with progressive retinal layer thinning and visual field loss within 2–4 years	Quaranta et al. [5] Rodrigo et al. [29]

## Data Availability

The data that support the findings of this study are available from the corresponding author upon request.
